# New evidence indicates the presence of barracuda (Sphyraenidae) and supports a tropical marine environment in the Miocene of Madagascar

**DOI:** 10.1371/journal.pone.0176553

**Published:** 2017-05-23

**Authors:** Michael D. Gottfried, Karen E. Samonds, Summer A. Ostrowski, Tsiory Harimalala Andrianavalona, Tolotra Niaina Ramihangihajason

**Affiliations:** 1 Department of Earth and Environmental Sciences, Michigan State University, East Lansing, Michigan United States of America; 2 Department of Biological Sciences, Northern Illinois University, DeKalb, Illinois, United States of America; 3 Department of Biological Sciences, University of Wisconsin-Parkside, Kenosha, Wisconsin, United States of America; 4 Département de Paléontologie et d’Anthropologie Biologique, Université d’Antananarivo, Antananarivo, Madagascar; University of California, UNITED STATES

## Abstract

Recent exploration of Miocene-age deposits at Nosy Makamby, a small island ~50 km southwest of Mahajanga city in northwestern Madagascar, has led to the recovery of a large sample [82] of isolated barracuda teeth (*Sphyraena* sp.). in a tropical marine fauna that also includes diverse marine invertebrates, chondrichthyans, bony fishes, turtles, crocodylians, and sirenians. Characteristically for barracudas, the teeth are labiolingually flattened and fang-like with a broadly triangular and blade-like acuminate outline and sharply edged but unserrated cutting margins. These barracudas inhabited an environment that included coral reefs (based on fossil scleractinians) and seagrass beds (evidenced by the epiphytic benthic foraminifera *Elphidium* sp.). The relatively common occurrence of Miocene barracuda at Nosy Makamby corroborates the presence of a tropical marine ecosystem encircling Madagascar by the Miocene, likely similar overall to the environment found there today.

## Introduction

Madagascar is perhaps the leading example of a ‘biodiversity hotspot’ owing to the exceptionally high rates of endemism seen in its native biota [1–3], and as such the deep-time evolutionary history of the island’s unique flora and fauna has long been of interest [4–8]. Field expeditions exploring a range of Mesozoic sites on the island have discovered some remarkable and highly informative fossils [9–16]; the Mesozoic finds are, however, unlikely to represent direct ancestors or very close relatives of the endemic forms that live on the island today. As noted by a number of researchers [4, 17, 18], Madagascar’s fossil history has a long gap that extends across much of the Cenozoic, from the early Paleocene up to the late Pleistocene and Holocene. Filling in this very sparse Cenozoic record is critical to interpreting the evolutionary history of Recent Malagasy taxa and the environmental conditions that accompanied their evolution.

**Fig 1:**
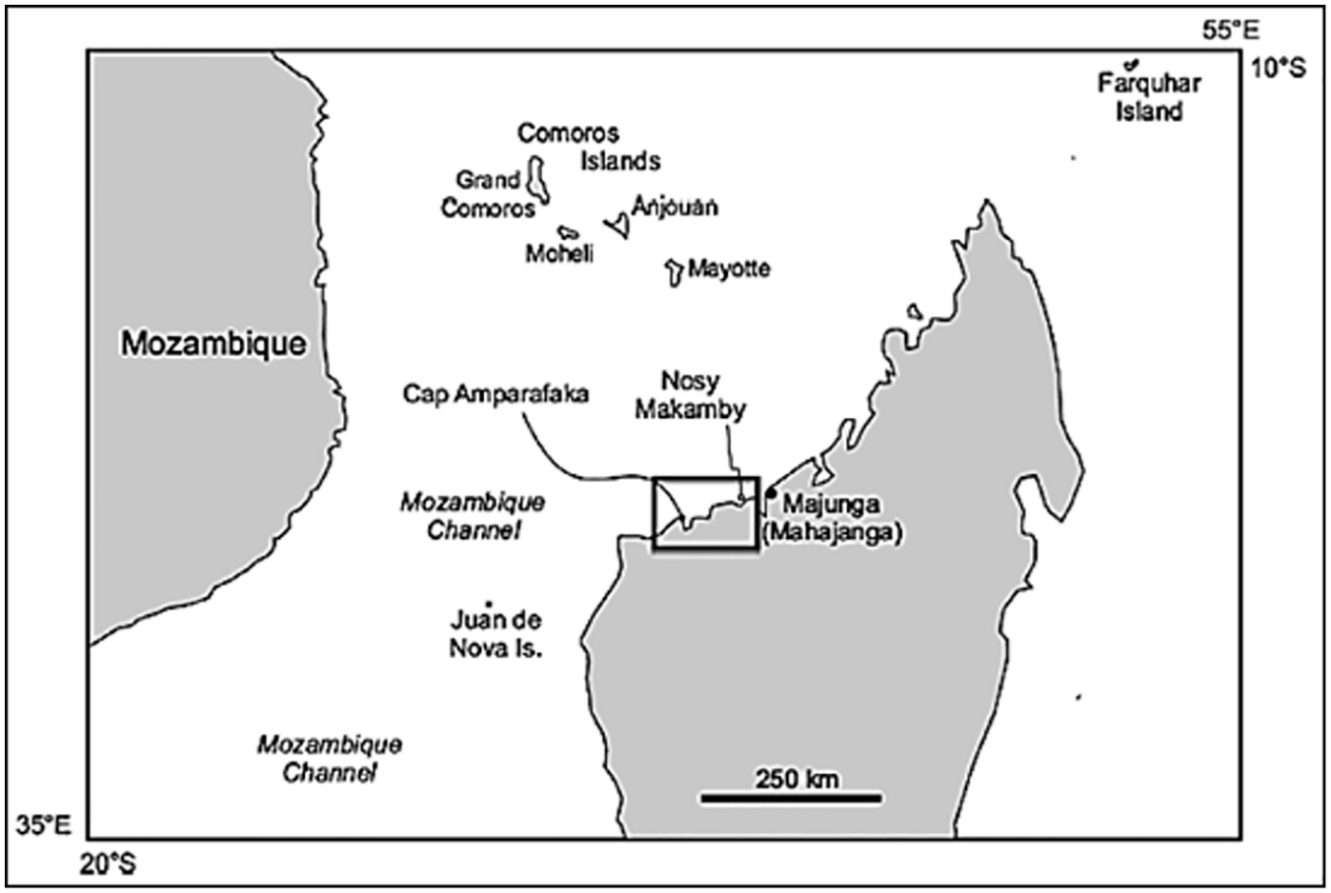
Location of Nosy Makamby off the northwestern coast of Madagascar.

**Fig 2:**
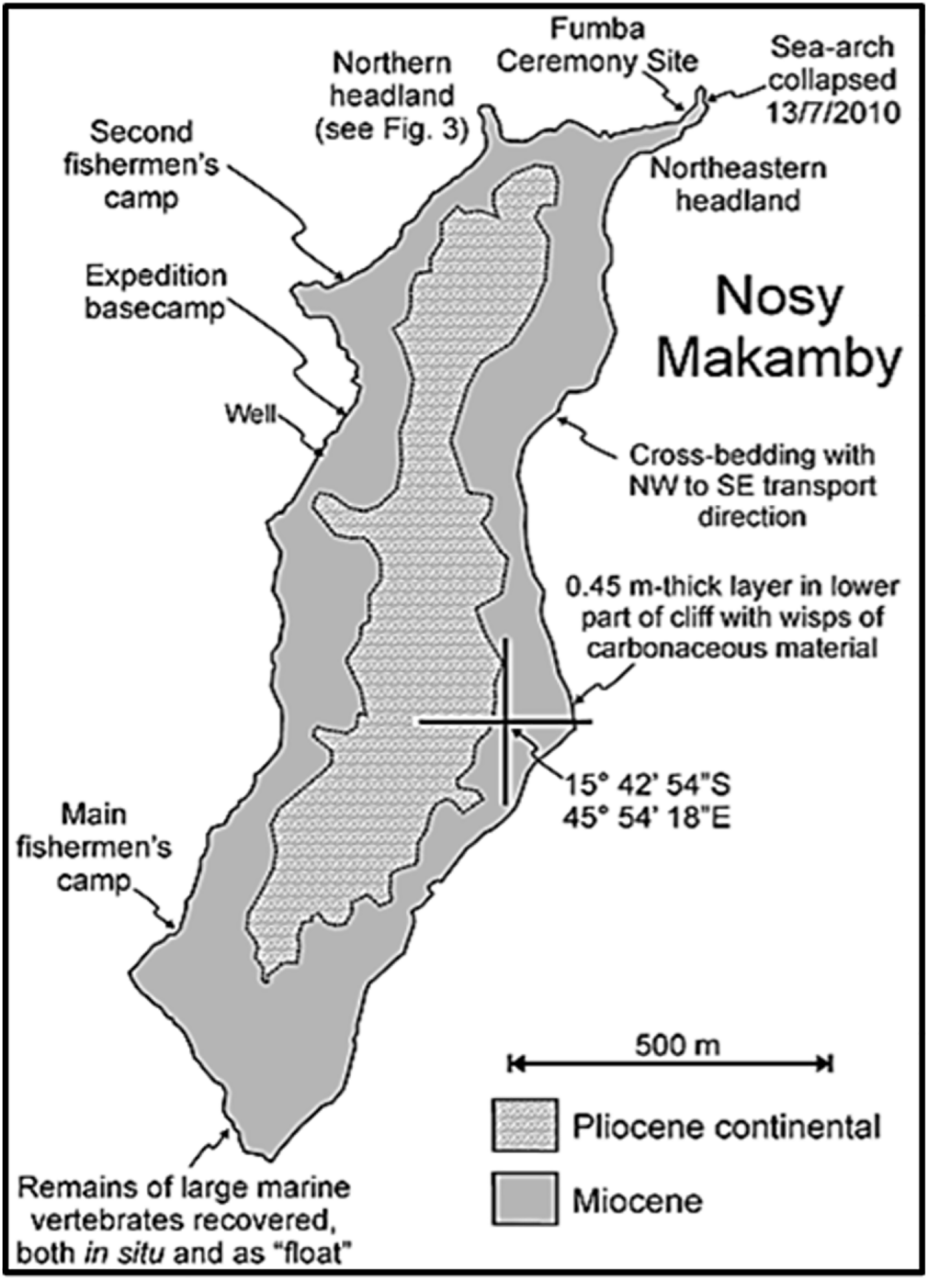
Nosy Makamby, showing location of principal Miocene fossil sites and geologic features.

**Fig 3:**
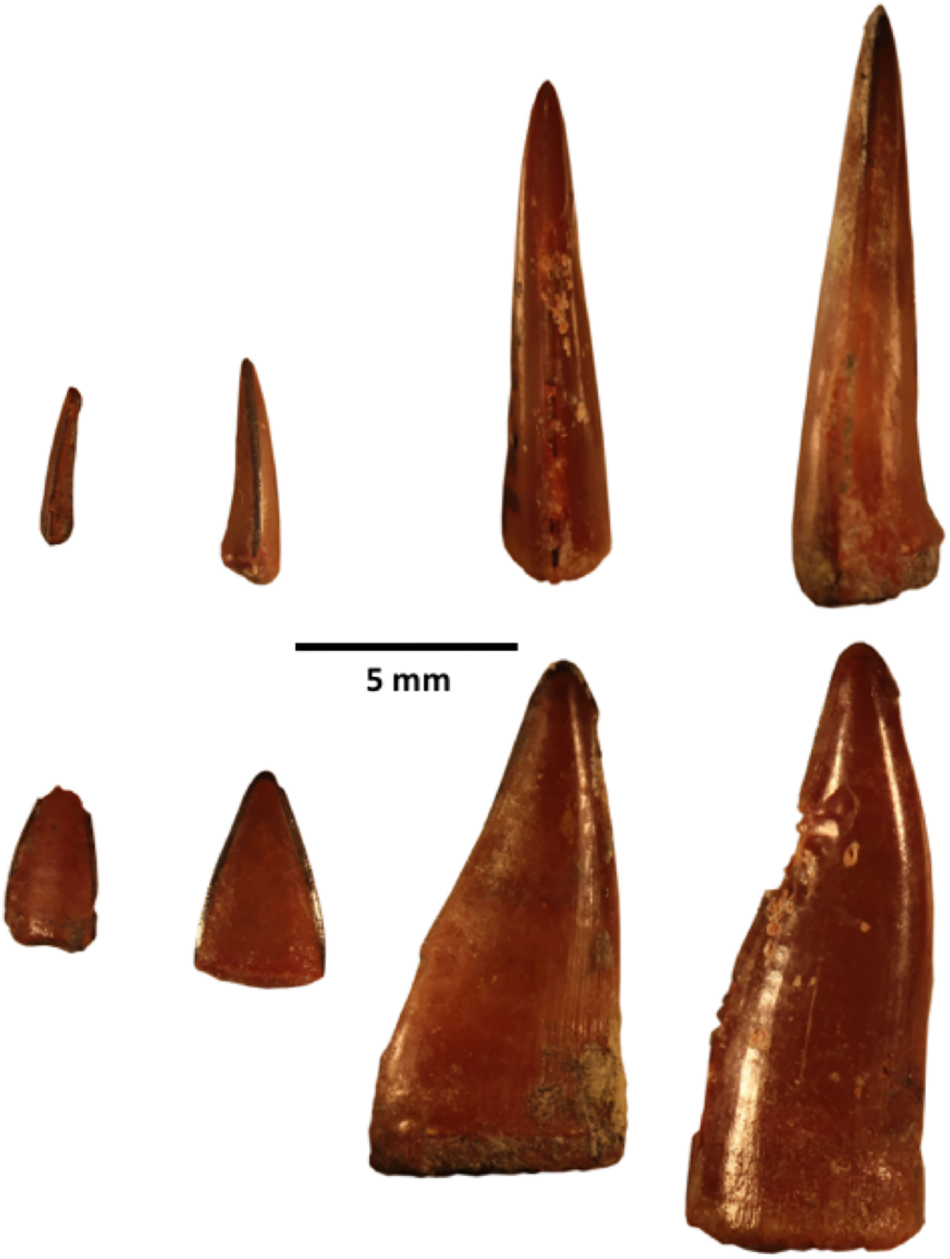
Fossil *Sphyraena* sp. teeth from Nosy Makamby. Upper row in side view, lower row shows same teeth rotated into labial/lingual view. Left to right, UA 10435, 10437, 10436, 11188.

Recent research [4, 19] has addressed closing this Cenozoic gap through a series of targeted expeditions. These investigations are also illuminating the broader paleoenvironment of Madagascar during this period, including the marine biota that surrounded the island. One specific site of interest is the small island of Nosy Makamby located along the coast of northwestern Madagascar ca. 50 km southwest of the city of Mahajanga (Figs 1 and 2). Andrianavalona et al. [19] recently described the Miocene shark and batoid fauna from Nosy Makamby, which includes 10 taxa of chondrichthyans, six of which were previously unreported from Madagascar. These inhabited a nearshore environment that also contained corals, bivalves, gastropods, bony fishes, turtles, crocodylians, and sirenians. Here we focus on the sphyraenid teleosts, more commonly known as ‘barracudas’–based on a large sample of teeth (see Fig 3) barracudas were commonly present, and (as adults) they would have occupied an apex predator niche in the marine ecosystem that encircled Madagascar in the Miocene.

**Fig 4:**
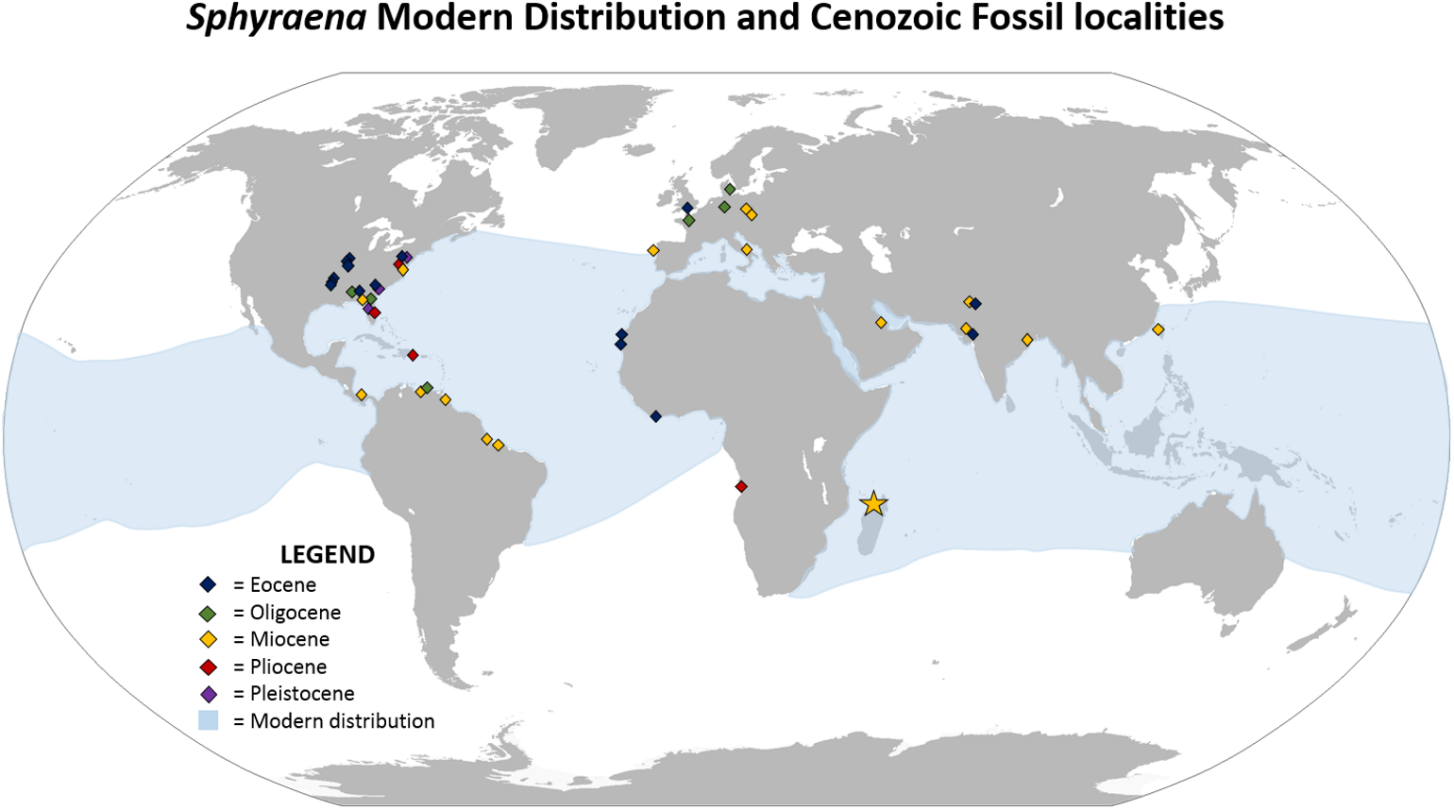
Recent distribution of barracudas (shown in light blue) and sites where fossil *Sphyraena* have been recovered.Yellow star indicates Nosy Makamby site. Some fossil distributional data obtained from Paleobiology Database [31].

Sphyraenids are acanthomorph teleosts traditionally placed within the scombroids (mackerels and allies). They are widely distributed as fossils during the Cenozoic, particularly in tropical to subtropical latitudes in the Atlantic Basin (see Fig 4). They have not been previously reported as fossils from Madagascar other than in a recent abstract; [20], but they are known from the Miocene of India [21] and the western Pacific [22]. The Family Sphyraenidae is represented by ~27 Recent species [23], which are found in the Atlantic, Pacific, and Indian oceans, where they are a conspicuous and ecologically significant large piscivore in coral reef and adjacent marine environments. Santini et al. [23] hypothesized that barracudas originated in the late Paleocene ca. 57 Ma, and subsequently radiated by the middle Eocene ca. 45 Ma. We document here a collection of 82 Miocene sphyraenid teeth from Nosy Makamby, northwestern Madagascar.

## Sphyraenid characteristics and affinities

Barracuda are morphologically distinctive, with elongate fusiform bodies, large gapes, an overshot lower jaw, and prominent fang-like labiolingually compressed teeth. Large individuals of the larger species can reach 2 m in total length, but most adults do not exceed ca. 1.5 m. They are often seen around scleractinian coral reefs, but are also know to occur in more open water and to cross open tracts of ocean [25], and to frequent seagrass beds as juveniles [26]. As adults they are typically opportunistic ambush predators on reef fishes, and may occur in schools of hundreds of individuals. Traditionally, sphyraenids were placed in Scombroidei, but recently it was hypothesized [27] that scombroids are non-monophyletic and that barracudas are putatively closer to carangids (jacks and pompanos), or centropomids (snooks and Nile perches), or possibly pleuronectiforms (flatfishes). Betancur-R. et al. [28] placed Sphyraenidae as *incertae sedis* in their Subseries Carangimorphariae, and we follow that classification here.

## Materials and methods

Fossils were obtained through surface collecting at sites on Nosy Makamby (15.7° S, 45.9° E) and from bulk sampling of matrix from productive horizons. Residue was broken down, prepared, sieved [with sieve sizes from 0.5–2.0 mm], and studied as outlined in Andrianavalona et al. [19]. All specimens included are catalogued into the collections of the Laboratory of Paleontology and Biostratigraphy, Department of Paleontology and Biological Anthropology, of the Université d’Antananarivo, Antananarivo, Madagascar (UAP). All necessary permits were obtained from the Malagasy Ministry of Mines for this study, in compliance with all relevant regulations. Specimens included in this report are on loan to KES at Northern Illinois University.

## Systematic paleontology

Superclass Actinopterygii

Infraclass Teleostei

Subsection Acanthomorphata

Series Carangimorpharia

Subseries Carangimorphariae

Family Sphyraenidae

*Sphyraena* sp.

### Referred specimens

Eighty-two teeth catalogued into the collections at the Université d’Antananarivo [Madagascar]: UAP 05379, 10220, 10242 [lot of 6], 10261, 10274, 10276, 10277, 10279–10283, 10292, 10297, 10314, 10324–10328, 10349–10352, 10358, 10359, 10377, 10378, 10381, 10384–10388, 10391–10396, 10431, 10434, 10435, 10437–10441, 10464, 10466, 10467, 10469, 10512, 10514, 10515, 11152, 11156, 11157, 11172 [lot of 7], 11186, 11203, 11211 [lot of 2], 11216, 11231, 11236 [lot of 2], 11246 [lot of 3], 11334.

### Comparative material examined

Natural History Museum (London): *Sphyraena lugardi* [Eocene, Nigeria] P.13736 (holotype); *Sphyraena cudmorei* [Miocene, Australia] P. 13975; *Sphyraena* sp. [Miocene, East Africa] P.12146, P.12621, P.14001, P.30152, -54, -55,-56, -57. American Museum of Natural History: *Sphyraena major* [Miocene, South Carolina] AMNH FF 3093, 9794, 10438, 10450; *Sphyraena bolcensis* [Eocene, Italy] AMNH FF 8000. Field Museum of Natural History: *Sphyraena* sp. [Oligocene, Mississippi] FMNH UF 965.

### Geologic age and setting

Recent investigations on Nosy Makamby have produced a relatively diverse Miocene fauna of invertebrate fossils that includes benthic foraminiferans, scleractinian corals, bivalve and gastropod mollusks, decapod arthropods, and echinoid echinoderms, and vertebrate fossils including chondrichthyans, teleost fishes, turtles and crocodylians, sirenians, bats, and one purported isolated rodent tooth [19, 24, 28]. The exposed section on Nosy Makamby includes a ca. 15 m thick sequence of Miocene clastics that consists of medium to coarse calcareous sandstones indicative of relatively high-energy deposition in a nearshore marine tropical setting. The Miocene beds include a distinctive ca. 2.5 m thick horizon that is heavily shot through with preserved tubes of the fossil ‘shipworm’ *Kuphus*, which is a useful marker bed that outcrops conspicuously around the island. The Miocene horizons are capped by a non-fossiliferous Pliocene continental red bed unit.

### Description

We base our identification of sphyraenid teleosts (barracudas) from Nosy Makamby on the relatively large sample of isolated teeth accumulated through surface collecting, and both wet and dry screening. To date 82 individual sphyraenid teeth have been recovered and catalogued, ranging in size from < 0.2 cm to nearly 2.0 cm in height (several dozen additional sphyraenid teeth were collected during the 2015 expedition but these are not yet available for study). Field collecting and matrix processing and sieving protocols can be found in Andrianavalona et al. [19].

Barracuda teeth are morphologically distinctive, and readily distinguished from other teeth (including sharks) that occur at Nosy Makamby (the Family Sphyraenidae is monogeneric, containing only *Sphyraena*). *Sphyraena* sp. teeth are triangular in outline shape, acuminate, unserrated along their cutting edges, and strongly labiolingually compressed. The bases of the teeth, when complete, are slightly constricted, which marks where they attach to the jaw, and they lack the strongly developed divergent roots characteristic of a variety of shark taxa that can have similar-looking crowns. The larger fang-like teeth that are borne on the anterior end of the gape (on the premaxilla and anterior dentary) are sigmoidal in outline, with the recurved tips of the teeth bent slightly posteriorly. Teeth of the Wahoo (*Acanthocybium solandri*), which also occurs widely in tropical marine environments [29], are similar to those of barracuda, but Wahoos lack the enlarged and sigmoidally curved fangs, a number of which have been recovered at Nosy Makamby. No jaw or other bony skeletal elements clearly attributable to barracudas have been recovered from Nosy Makamby, but the collection of teeth taken as a whole is diagnostic for the presence of *Sphyraena* and strong evidence that barracuda were common in the Miocene marine ecosystem on the western side of Madagascar.

## Discussion

Santini et al. [23] maintained on the basis of molecular data that sphyraenids originated in the late Paleocene ca. 57 Ma and subsequently radiated in the Eocene, with fossil members of the extant lineages appearing by the Miocene. The Nosy Makamby fishes are consistent with this hypothesis. They could represent either late-surviving relatives of the earlier lineages, or early members of one of the extant species groups. Eleven extant species of barracuda have been reported from the western Indian Ocean, but because our sample consists solely of isolated teeth, without additional skeletal material preserved that would facilitate more detailed morphological comparisons, we are not able to suggest a species-level assignment or an assertion that the teeth are more closely related to any one particular extant species of *Sphyraena*.

The relative abundance of *Sphyraena* sp. teeth at Nosy Makamby indicates that these large piscivores were common in the tropical Miocene seas around Madagascar, and along with the macrophagous sharks they would have occupied an apex position in the marine food chain. In addition to the presence of barracudas, a tropical environment in the Miocene of Madagascar is strongly indicated by the overall fauna that has been recovered from Nosy Makamby, which considered in combination includes tropical invertebrates [24, 30], chondrichthyans [19], and porcupine fish [19]. The Miocene marine ecosystem in the Mozambique Channel along the west coast of Madagascar was likely broadly similar to the tropical environment that exists there today.

Recent work by Ramihangihajason et al. [24] indicates that epiphytic foraminiferans (*Elphidium*) associated with seagrass beds were part of the Nosy Makamby Miocene environment. Juvenile Recent barracuda often develop in seagrass beds [26] where they are sheltered and not as subject to predation by larger fishes, including adult barracudas. It is possible that this ecological relationship existed by the Miocene, with small and relatively vulnerable barracudas taking refuge in the seagrass around present-day Nosy Makamby until they were older and large enough to escape predation and be successful reef and open water predators.
